# Optimal timing of vitrectomy for severe mechanical ocular trauma: A retrospective observational study

**DOI:** 10.1038/s41598-019-54472-9

**Published:** 2019-11-29

**Authors:** Honghua Yu, Jianhua Li, Ying Yu, Guodong Li, Dongli Li, Meng Guan, Li Lu, Ting Liu, Yujuan Luo, Lu Shen, Qiaowei Wu, Baoyi Liu, Songfu Feng, Ling Yuan

**Affiliations:** 1 Department of Ophthalmology, the First Affiliated Hospital of Kunming Medical University, Kunming, 650031 China; 2 Department of Ophthalmology, Guangdong Eye Institute, Guangdong Provincial People’s Hospital, Guangdong Academy of Medical Sciences, Guangzhou, 510080 China; 3 Department of Ophthalmology, Guangzhou Women and Children’s Medical Center, Guangzhou Medical University, Guangzhou, 510230 China; 4 Department of Ophthalmology, Zhujiang Hospital, Southern Medical University, Guangzhou, 510280 China; 5 Department of Ophthalmology, the Second People’s Hospital of Jiangxi, Nanchang, 330000 China

**Keywords:** Outcomes research, Prognosis

## Abstract

Mechanical ocular trauma could lead to disastrous visual outcomes. There has been a controversy regarding the timing of vitrectomy for such cases. This study aimed to find out the optimal timing of vitrectomy for severe mechanical ocular trauma. Patients with severe mechanical ocular trauma who had undergone vitrectomy were enrolled and followed up for at least 6 months. Clinical data were collected including ocular trauma score (OTS), the timing of vitrectomy upon injury, visual acuity, vitrectomy results, post-operation complications and etc. All cases were classified according to the timing of vitrectomy upon injury into 3 groups: group A 1–7 days, group B 8–14 days, group C more than 14 days. A total of 62 cases were enrolled, including 20 eyes in group A, 25 eyes in group B, and 17 eyes in group C. No significant differences were shown of the gender, age or OTS among the 3 groups. Both functional success rate and visual outcome were optimal in group B, then in group A, and worst in group C. These results suggested that the best timing of vitrectomy for severe mechanical ocular trauma is 8–14 days upon injury; second best is 1–7 days; worst is after 14 days.

## Introduction

Ocular trauma could vary from mild injuries that only requires topical antibiotics to vision-threatening trauma that requires prompt surgical intervention^1^. One of the most common causes of unilateral blindness is trauma, which also accounts for the bilateral blindness of more than one million people around the world^2,3^. Mechanical ocular trauma is the result of striking the eye or periocular tissues with blunt or sharp objects, in exclusion of electrical, chemical or thermal injuries^4^. Most of mechanical ocular trauma occur in the workplace or during recreational activities, mostly occur in males, and could lead to disastrous visual outcomes^5^.

Severe mechanical ocular trauma has been subdivided into open-globe (eye rupture, penetration injury, and intraocular foreign body) and closed-globe injuries (retinal detachment, macular hole, and vitreous hemorrhage)^4^. Both types of injuries may be indications for vitrectomy^6^.

Vitrectomy could restore certain visual acuity by restoring the transparency of the refractive media, repositioning the retina, and saving the injured eye^7^. Prompt vitrectomy is indicated in patients with intraocular foreign body or endophthalmitis. However, the timing of vitrectomy for patients with traumatic retinal detachment or vitreous hemorrhage is still controversial.^8,9^. Therefore, the present study was designed to find out the optimal timing of vitrectomy for severe mechanical ocular trauma.

## Results

### General information

62 patients were enrolled in this study including 51 male (82.3%) and 11 female (17.8%), aging from 10–69 years old, with an average of 36.6 ± 12.8 years old. There was no statistically significant difference of gender or age among three groups (all P > 0.05) (Table 1). All patients enrolled were with unilateral severe mechanical ocular trauma.

**Table 1:** General information among the groups.

	Group A* (n = 20)	Group B* (n = 25)	Group C* (n = 17)	X^2^/F value*	P value
Gender (M/F)	16/4	21/4	14/3	0.122	0.941
Age (mean ± SD)	40.4 ± 15.1	37.5 ± 12.8	31.8 ± 11.6	1.971	0.148
OTS (mean ± SD)	53.9 ± 12.5	53.5 ± 15.6	53.9 ± 16.4	0.005	0.995
Contusion, n (%)	6 (30.0)	8 (32.0)	4 (23.5)	0.366	0.833
Perforating injury, n (%)	9 (45.0)	15 (60.0)	12 (70.6)	2.535	0.281
Rupture, n (%)	5 (25.0)	12 (48.0)	0 (0.0)	11.802	0.003**
Penetrating injury, n (%)	0 (0.0)	0 (0.0)	1 (5.9)	2.690	0.260

Abbreviation: M, male; F, female; OTS, ocular trauma score; n, number.

*Group A: Vitrectomy was performed on the 1^st^–7^th^ day upon injury.

*Group B: Vitrectomy was performed on the 8^th^−14^th^ day upon injury.

*Group C: Vitrectomy was performed after 14 days upon injury.

*X^2^/F: Chi-square test was applied to compare the differences of gender and incidences of contusion, perforating injury, rupture and penetrating injury among the three groups. One-way ANOVA test was applied to compare the differences of age and OTS among the three groups.

**P < 0.05.

According to the timing of vitrectomy upon injury, all cases were classified into three groups. Group A included 20 eyes on which vitrectomy was performed 1–7 days after injury. Group B included 25 eyes on which vitrectomy was performed 8–14 days after injury. Group C included 17 eyes on which vitrectomy was performed after 14 days after injury.

According to the classifying system of mechanical injuries of the eye^4^, there were 44 cases (71%) of open-globe injuries, 18 cases (29%) of closed-globe injuries in this study. To be specific, there were 18 cases (29.0%) of contusion, 36 cases (58.1%) of perforating injuries, 7 cases (11.3%) of rupture, 1 case (1.6%) of penetrating injury. The causes of these injuries include iron wire or nail (25 cases), stone (9 cases), firecracker (4 cases), rubber (3 cases), glass (2 cases) and others (19 cases). There was no statistically significant difference of types of the injuries among three groups (all P > 0.05), except for rupture (Table 1).

All cases in the study had undergone vitrectomy. Other procedures during surgery include lensectomy (36 cases), scleral buckling (2 cases), peeling of epiretinal membrane (9 cases), silicone oil filling (31 cases), C3F8 injection (13 cases) and retinal photocoagulation (49 cases).

### Comparing ocular trauma score (OTS) before surgery

OTS is an indicator of the severity of ocular trauma, clinically used to predict the visual outcome of patients after ocular trauma^10^. The OTS in group A ranged from 35–76, group B ranged from 25–76, group C ranged from 25–81. There was no statistically significant difference of OTS between each two groups (all P > 0.05) (Table 1).

### Comparing vitrectomy results after surgery

According to Ryan *et al*.’s classifying method for vitrectomy results^11^, all cases were classified into functional success, anatomic success and failure. Functional success was improvement in visual acuity of two lines on the Snellen chart; improvement from light perception or hand motions to 3/90 (5/200); and maintenance of good preoperative vision. Anatomic success was those cases in which the eye was anatomically rehabilitated, but there was no functional improvement for reasons other than successful surgery.

The results of our study showed that the functional success rate was highest in group B (96.0%), then in group A (85.0%), and lowest in group C (52.9%) (Table 2). There was a significant difference of functional success rate among the three groups (P = 0.002).

**Table 2:** Vitrectomy results and complications distribution among the groups (number of cases).

	Group A* (n = 20)	Group B* (n = 25)	Group C* (n = 17)	Total (n = 62)	X^2^ value*	P value
Functional Success, n (%)	17 (85.0)	24 (96.0)	9 (53.0)	50 (80.7)	12.378	0.002**
Anatomical Success, n (%)	3 (15.0)	1 (4.0)	6 (35.3)	10 (16.1)	7.353	0.025**
Failure, n (%)	0 (0.0)	0 (0.0)	2 (11.8)	2 (3.2)	5.471	0.065
Second vitrectomy, n (%)	2 (10.0)	1 (4.0)	3 (17.6)	6 (9.7)	2.160	0.340
Silicone oil dependent, n (%)	0 (0.0)	0 (0.0)	2 (11.8)	2 (3.2)	5.471	0.065
Corneal leukoma, n (%)	1 (5.0)	2 (8.0)	2 (11.8)	5 (8.1)	0.567	0.753
High IOP, n (%)	2 (10.0)	4 (16.0)	1 (5.9)	7 (11.3)	1.083	0.582

Abbreviation: n, number; IOP, intraocular pressure.

*Group A: Vitrectomy was performed on the 1^st^–7^th^ day upon injury.

*Group B: Vitrectomy was performed on the 8^th^–14^th^ day upon injury.

*Group C: Vitrectomy was performed after 14 days upon injury.

*X^2^/F: Chi-square test was applied to compare the differences among the three groups.

**P < 0.05.

### Comparing Visual acuity before and after surgery

**Figure 1:**
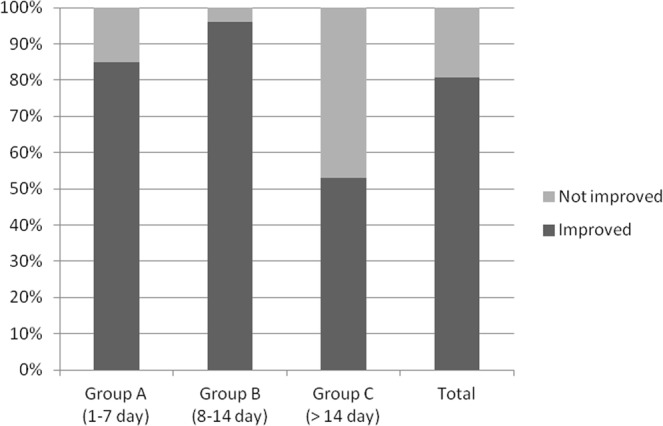
Percentage of cases with an improvement of visual acuity after vitrectomy. After vitrectomy, 50 eyes (80.7%) had acquired improvement of visual acuity, among which group A includes 17 cases (85.0%), group B includes 24 cases (96.0%), group C includes 9 cases (52.9%), in concordance with the distribution of functional success rate.

Before surgery, there was no statistically significant difference of visual acuity among the three groups (P = 0.740) (Table 3). After vitrectomy, only 5 eyes (8.06%) had recovered to their visual acuity before the injuries. However, comparing with the visual acuity before surgery, 50 eyes (80.7%) had acquired improvement of visual acuity, among which group A includes 17 cases (85.0%), group B includes 24 cases (96.0%), group C includes 9 cases (52.9%), in concordance with the distribution of functional success rate (Fig. 1). The post-operative visual acuity was significantly improved compared to the initial visual acuity (P < 0.001). There was also a significant difference of visual acuity after surgery among the three groups (P = 0.045).

**Table 3:** Visual acuity distribution among the groups (number of cases).

		≥20/40	20/50-20/100	19/100-5/200	4/200-LP*	NLP*	P value*
Total		9	11	26	15	1	
Before surgery	Group A	0	1	2	16	1	0.740
	Group B	0	2	1	19	3	
	Group C	0	1	0	15	1	
After surgery	Group A	3	3	10	4	0	0.045**
	Group B	5	5	12	3	0	
	Group C	1	3	4	8	1	

Abbreviation: LP, light perception; NLP, no light perception.

*Group A: Vitrectomy was performed on the 1^st^–7^th^ day upon injury.

*Group B: Vitrectomy was performed on the 8^th^–14^th^ day upon injury.

*Group C: Vitrectomy was performed after 14 days upon injury.

*P value: Chi-square test was applied to compare the differences of visual acuity among the three groups.

**P < 0.05.

### Post-operation complications

All patients were followed up for more than 6 months, up to 22 months, with an average of 11.5 ± 3.4 months. No endophthalmitis or atrophy of eyeball were observed. A total of 45 eyes had had retinal detachment before surgery. Thirty-nine of them were successfully restored to their position after the first vitrectomy. Six of them had to go through the second vitrectomy, including 2 cases in group A, 1 case in group B and 3 cases in group C (Table 2). Two of group C became silicone-oil-dependent after the third vitrectomy. All cases had corneal edema in varying degrees after surgery. Most of the corneas regained the transparency after 5–7 days of treatment. Five eyes developed corneal leukoma. High intraocular pressure (IOP) occurred in 7 eyes after surgery. There was no statistically significant difference of above complications among three groups (all P > 0.05) (Table 2). Six of them restored to normal IOP after anti-glaucoma treatment. The other 1 eye had to receive trabeculectomy. And the IOP stayed in control since. Two eyes became silicone-oil-dependent as the silicone oil could not be removed for the sake of proliferative vitreoretinopathy.

## Discussion

Vitrectomy is an important treatment for severe mechanical ocular trauma^12,13^. It was reported that 28% of open-globe injuries require eyeball enucleation^14^. However, with the continuous development of vitreoretinal surgery technique, 23 G, 25 G, 27 G minimally invasive vitrectomy has brought light to the treatment of severe mechanical ocular trauma, greatly reducing the rate of eyeball enucleation^15,16^. The results of our study showed that most eyes with mechanical trauma could be saved and restored a certain visual function after treated with vitrectomy. Fifty out of 62 cases (80.7%) achieved an improvement in visual acuity with functional success as a result of vitrectomy.

Nevertheless, one of the challenges in the treatment of severe mechanical ocular trauma is identifying the optimal timing for vitrectomy^17^. There has been a controversy about the best timing of vitrectomy for ocular trauma^8,9^.

Some studies have shown less importance of vitrectomy timing to the outcomes of ocular trauma. A study led by Mansouri *et al*. showed that the timing of vitrectomy was not associated with the visual outcome among 90 patients with ocular injuries involving the posterior segment^18^. Similarly, Ghoraba *et al*. analyzed 207 eyes of 197 patients with gunshot perforating eye injury and demonstrated that, there was no significant difference between the two groups (3–4 weeks and after 4 weeks) in either anatomical or functional results, but suggested the vitrectomy be done before the 5^th^ week, as retinal detachment was more likely to occur on eyes operated after the 4^th^ week^19^.

However, some studies supported early vitrectomy. Chen *et al*. studied 120 patients with open-globe injury with traumatic optic neuropathy whom were divided into two groups (vitrectomy performed within 1 week and after 1 week upon injury) and showed that early vitrectomy could augment the visual acuity, decrease complications, improve prognosis and reversibly affect the thickness of retinal nerve fiber layer^20^.

Different with the above studies, the results of our study demonstrated that the best timing of vitrectomy for severe mechanical ocular trauma is the 8^th^ to 14^th^ day upon injury; second best is the 1^st^ to 7^th^ day; worst is after 14 days. Both functional success rate and visual outcome were optimal in patients who had vitrectomy in the 8^th^ to 14^th^ day upon injury. These results are in accordance with the conclusion from Kuhn’s review, which is among the 4 stages (2–4 days, 5–7 days, 8–14 days and past 2 weeks) to perform vitrectomy for patients with severe mechanical trauma, earlier stages showed higher risk of intraoperative complications, while later stages showed higher incidence and severity of postoperative complications such as proliferative vitreoretinopathy, the most damaging kind^21^.

Early vitrectomy on acutely injured eyes could bring higher risk of continuous hemorrhage. Vitreoretinal surgery performed within one week after trauma would be more challenging as the spontaneous posterior vitreous detachment (PVD) had not been formed yet. During 8 to 14 days after injury, the originally lacerated vessels were healed and the inflammation was alleviated. The fibroproliferative membranes were still easy to peel and cut during this time, and a PVD would have usually formed by then. For group C, the vitrectomy was performed after two weeks upon injury, fibrovascular proliferation and inflammation could often lead to contraction at this stage.

Proliferative vitreoretinopathy (PVR) is a common complication of ocular trauma. The possible risk factors for developing PVR includes intraoperative findings of retinal stiffness, vitreous cavity mixed with big amount of pigment, younger patients, large retinal tear and other risk factors. The time from ocular trauma to the onset of PVR ranges from 1 to 6 months^22^. The inflammatory reaction that follows injury could bring the immune cells into the vitreous cavity, which could stimulate the production of growth factors and cytokines, promoting an environment of cell proliferation, migration and differentiation. Contraction of these proliferative membranes could have disastrous consequences for vision.

Results showed that 6 cases had to go through the second vitrectomy. The reasons for this were the residual peripheral vitreous in a young patient and the unclosed large retina tear in the other case in group A; the unclosed large retina tear in 1 case in group B; the PVR development in 2 cases and the residual peripheral vitreous in a young patient in group C. The reason why 2 cases became silicone-oil-dependent after the third vitrectomy in group C could also be the development of PVR.

No statistically significant differences were shown of the gender, age or OTS among the three groups, which could eliminate the bias of the results to some extent. Most studies have recommended the prompt removal of the intraocular foreign bodies, within 24–48 hours after trauma, because of the risk of endophthalmitis and toxic reactions^23–25^. Thus we have excluded such cases to avoid the bias.

However, there were some limitations of this study. First, the size of sample and length of follow-up were limited. As many patients with ocular trauma are low-income workers, their follow-up compliance is relatively poor, so follow-up will be harder to conduct when a longer time or more times of follow-up is required. Second, the study cohort was mostly comprised of the open-globe injury cases and a low percentage of penetrating injury, which may lead to selection bias. Third, the OTS might be similar among treatment groups having lower visual acuity due to vitreous hemorrhage before surgery. In addition, the silicone oil was not removed in some of the cases at the endpoint, which could have potential effect afterwards. More studies with larger sample size and longer follow-up are needed in the future to investigate the optimal timing of vitrectomy for severe mechanical ocular trauma.

In conclusion, our study showed that both functional success rate and visual outcome were optimal in patients who had vitrectomy in the 8^th^ to 14^th^ day upon injury, suggesting that the best timing of vitrectomy for severe mechanical ocular trauma is the 8^th^ to 14^th^ day upon injury; second best is the 1^st^ to 7^th^ day; worst is after 14 days.

## Methods

### Study cohort

This retrospective observational study was approved by the Ethics Committee of the First Affiliated Hospital of Kunming Medical University (Kunming, China) and adhered to the tenets of the Declaration of Helsinki. Informed consents were obtained from all individual participants for study participation. The study cohort was recruited from the patients who were hospitalized in the ophthalmology department of the First Affiliated Hospital of Kunming Medical University during June 2015 to September 2016. All data were anonymized and de-identified before analysis.

Patients with severe mechanical ocular trauma who had undergone vitrectomy were enrolled in this study and followed up for at least 6 months. To be specific, the inclusion criteria were the patients with injury of the posterior segment of the eye with indications for vitrectomy, which includes at least one of the following signs observed before or during operation: (1) vitreous hemorrhage or opacity; (2) retinal detachment, retinal tear, or macular hole; (3) choroidal detachment; (4) rupture of retina and choroid; (5) subretinal hemorrhage. The exclusion criteria were: (1) patients with incomplete data; (2) patients with intraocular foreign bodies, traumatic endophthalmitis or any other situation that required emergency vitrectomy (Since selective surgery is not an option, they are excluded from the study); (3) patients with any congenital eye diseases.

### Collections of clinical data

Clinical data were collected including age, gender, diagnosis, injury causes, injury type, OTS^26^, the timing of vitrectomy upon injury, visual acuity, vitrectomy results, post-operation complications, condition of the anterior segment and fundus, ultrasonic test results of the vitreous and retinal condition, CT examination results for those who were suspected of bearing intraocular foreign bodies. OTS scores range from 1 as most severe injury to 5 as least severe injury.

### Grouping methods

The included eyes were classified according to the timing of vitrectomy upon injury into three groups: group A 1–7 days, group B 8–14 days, group C more than 14 days. Poor visual function is mainly related to retinal detachment caused by trauma itself, corneal scar healing, possible optic nerve injury, damage caused by surgery itself, and toxicity of intraocular fillers such as silicone oil or gas. Ocular injuries caused by trauma are usually complex injuries, and it is difficult to classify and compare the injuries in a single aspect. Therefore, we classified them according to the timing of surgical treatment after injury.

### Surgery methods

The levofloxacin eye drops were routinely administered before surgery. Anti-infection and hemostasis treatments were applied when necessary. Primary wound closure was performed on patients with open-globe ocular trauma. The pars plana vitrectomy was performed using the Alcon 23-gauge micro-vitrectomy system (Alcon Laboratories Inc, USA.). The three channels were created at the superior temporal, infratemporal, and superior nasal position. Catheter, infusion tube, and vitrectomy head were placed. According to the ocular condition, first remove the cloudy lens and inflammatory exudates in the anterior chamber, then remove the cloudy vitreous and hemorrhage. Perfluorocarbon liquids were used if necessary, to reduce bleeding in the posterior segment, press the retina, and divest the retinal proliferating membrane. For twisted, adherent and rotated retina tissue, retinotomy was performed. The subretinal fluid was sucked out from the original retinal tear. For patients with choroidal hemorrhage, the blood was drained through the sclera puncture. Retinal laser photocoagulation was applied on the periphery and degenerate area of the retinal tear, and covering 360° if necessary. After the exchange of gas and liquid, silicone oil or gas was injected depending on the retinal condition. When the intraocular pressure was restored, the scleral incisions were closed and the bulbar conjunctiva tissues were oversewn. After the surgery, erythromycin eye ointment was covered on the eye.

### Post-operative treatments

After operation, the patients were set into prone position. Antibiotic treatment was applied locally, and systematically when necessary. For patients with severe inflammation, dexamethasone was injected. After discharge, local treatment was applied for two weeks. Silicone oil was removed in the 3^rd^ to 12^th^ month after operation. Second-stage implantation of intraocular lens was performed depending on the retinal condition.

### Follow-ups

All patients in this study were followed-up on the 1^st^ week, 1^st^ month, 3^rd^ month and 6^th^ month after the surgery. Best corrected visual acuity (BCVA) and IOP were tested. The complications and retinal reposition progress were monitored. The frequency of follow-up could be raised according to the condition of the eye. Each follow-up was examined and evaluated by the same doctor.

### Data analysis

SPSS 20.0 statistics software was used to analyze the data. The continuous variables were presented as mean ± standard deviation (SD). One-way ANOVA test were applied for independent continuous variables. Chi-square test was applied for categorical variables. Mann-Whitney U test was applied for two independent rank variables. Kruskal–Wallis H test was applied for three independent rank variables. P < 0.05 was considered statistically significant.
